# Fine particulate matter air pollution and aortic perivascular adipose tissue: Oxidative stress, leptin, and vascular dysfunction

**DOI:** 10.14814/phy2.14980

**Published:** 2021-07-29

**Authors:** Petra Haberzettl, Lexiao Jin, Daniel W. Riggs, Jingjing Zhao, Timothy E. O’Toole, Daniel J. Conklin

**Affiliations:** ^1^ Diabetes and Obesity Center University of Louisville Louisville KY USA; ^2^ Christina Lee Brown Envirome Institute University of Louisville Louisville KY USA; ^3^ Division of Environmental Medicine University of Louisville Louisville KY USA

**Keywords:** acrolein, cardiovascular disease, endothelial dysfunction, environmental cardiology, PM_2.5_

## Abstract

Exposure to fine particulate matter (PM_2.5_) air pollution increases blood pressure, induces vascular inflammation and dysfunction, and augments atherosclerosis in humans and rodents; however, the understanding of early changes that foster chronic vascular disease is incomplete. Because perivascular adipose tissue (PVAT) inflammation is implicated in chronic vascular diseases, we investigated changes in aortic PVAT following short‐term air pollution exposure. Mice were exposed to HEPA‐filtered or concentrated ambient PM_2.5_ (CAP) for 9 consecutive days, and the abundance of inflammatory, adipogenic, and adipokine gene mRNAs was measured by gene array and *qRT*‐*PCR* in thoracic aortic PVAT. Responses of the isolated aorta with and without PVAT to contractile (phenylephrine, PE) and relaxant agonists (acetylcholine, ACh; sodium nitroprusside, SNP) were measured. Exposure to CAP significantly increased the urinary excretion of acrolein metabolite (3HPMA) as well as the abundance of protein–acrolein adducts (a marker of oxidative stress) in PVAT and aorta, upregulated PVAT *leptin* mRNA expression without changing mRNA levels of several proinflammatory genes, and induced PVAT insulin resistance. In control mice, PVAT significantly depressed PE‐induced contractions—an effect that was dampened by CAP exposure. Pulmonary overexpression of extracellular dismutase (ecSOD‐Tg) prevented CAP‐induced effects on urinary 3HPMA levels, PVAT *Lep* mRNA, and alterations in PVAT and aortic function, reflecting a necessary role of pulmonary oxidative stress in all of these deleterious CAP‐induced changes. More research is needed to address how exactly short‐term exposure to PM_2.5_ perturbs PVAT and aortic function, and how these specific genes and functional changes in PVAT could lead over time to chronic inflammation, endothelial dysfunction, and atherosclerosis.

## Graphical abstract

Short‐term (9‐day) exposure of mice to concentrated ambient particulate matter (CAP) alters the function of perivascular adipose tissue (PVAT) and aorta. Short‐term CAP exposure increased PVAT oxidative stress and leptin mRNA. Normally, PVAT and endothelium are anticontractile and modulate aortic vascular smooth muscle (VSMC) tone. After CAP exposure, however, the aorta becomes more contractile with accompanying loss of endothelial‐dependent modulation and increased PVAT‐dependent anticontractile function. The increased PVAT leptin may represent a compensatory response to maintain vascular tone balance. The CAP‐induced alterations in pulmonary and systemic oxidative stress, *leptin* mRNA abundance in PVAT, and aortic contractility were prevented in mice overexpressing pulmonary extracellular superoxide dismutase (ecSOD‐Tg) implicating pulmonary oxidative stress in CAP‐induced changes.

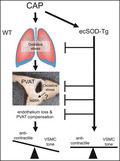

## 1 INTRODUCTION

Cardiovascular disease (CVD) remains the number one cause of morbidity and mortality in the world. Air pollution is the eighth leading cause of mortality worldwide contributing significantly to overall CVD mortality (Cohen et al., 2005). From prospective human cohort studies, case–controlled studies, panel exposure studies, and meta‐analyses as well as numerous controlled exposure studies in animals, there is significant evidence of the deleterious effects of PM_2.5_ on the cardiovascular system (Brook et al., 2010). Although these studies indicate an important role of inflammation in the effects of PM_2.5_ on CVD, the specific mechanisms by which acute exposures to PM_2.5_ alter vascular function are uncertain.

Chronic air pollution exposure of mice appears to activate the perivascular adipose tissue (PVAT), which surrounds blood vessels, by enhancing both the formation of reactive oxygen species (ROS) and the homing of inflammatory cells into the PVAT (Kampfrath et al., 2011). A similar process also is thought to occur in models of vascular inflammation, including diet‐induced obesity, that foster vascular dysfunction colloquially referred to as “outside‐inside remodeling” (Blomkalns et al., 2010; Chatterjee et al., 2009; Ketonen et al., 2010). It is hypothesized that proinflammatory changes in PVAT precede and are causal in endothelial dysfunction and the promotion of atherosclerosis and aortic aneurysm (Daugherty et al., 2011; Kim et al., 2020). Despite these associations, the relative role of PVAT in the early onset of air pollution‐induced vascular dysfunction is unclear (Knudson et al., 2008; Payne et al., 2014).

Physiologically, intact PVAT has a dominant anticontractile activity (Lohn et al., 2002). Adipocytes (brown and white) contribute to the majority of PVAT mass, and thus, adipokines, including adiponectin, leptin, and resistin among other adipose‐derived relaxing factors (ADRF), regulate both smooth muscle and endothelial cell function (Lohn et al., 2002; Rajsheker et al., 2010). Alterations in the cellular and inflammatory composition of PVAT under conditions of chronic high‐fat diet feeding (Chatterjee et al., 2009; Ketonen et al., 2010; Marchesi et al., 2009) as well as chronic air pollution exposure (Kampfrath et al., 2011) have been observed. In addition, PVAT transplant studies substantiate that proinflammatory PVAT releases vasoregulatory compounds that dysregulate vascular function (Blomkalns et al., 2010; Payne et al., 2010). For example, in addition to being an anticontractile adipokine, leptin also induces insulin resistance in brown adipocytes in culture (Kraus et al., 2002).

Although PVAT (composition and function) is altered in chronic air pollution and in metabolic disease states in humans and animal models (Payne et al., 2012; Withers et al., 2011), the potential role of PVAT in vascular changes after short‐term air pollution exposure has not been addressed. For example, exposure of mice to concentrated ambient PM_2.5_ (CAP) for only 9 days induces aortic inflammation and insulin and VEGF resistance (Haberzettl et al., 2012, 2016a,b), yet the contribution of PVAT to these changes is unknown. Thus, our study investigated the early changes in PVAT and aortic function following short‐term exposures of mice to CAP. Furthermore, we tested whether CAP‐induced PVAT and vascular changes were mitigated by selective pulmonary overexpression of extracellular superoxide dismutase (ecSOD) (Haberzettl et al., 2018; Haberzettl, O'Toole, et al., 2016).

## 2 MATERIALS AND METHODS

### 2.1 Mice

Mice were treated according to the APS’s Guiding Principles in the Care and Use of Animals and all protocols were approved by the University of Louisville IACUC. Male C57BL/6J mice (12 weeks old, Jackson Laboratory) fed a normal chow diet (NC, 13% kcal fat, LabDiets, Cincinnati, OH) were exposed for 9 days to HEPA‐filtered air or CAP. No food or water was provided during the daily (6 h) exposures. Additionally, mice overexpressing lung‐specific extracellular superoxide dismutase (ecSOD‐Tg) and their wild‐type (WT) littermates were exposed to air or CAP for 9 consecutive days. CAP (PM_2.5_ fraction) was concentrated (up to 10‐fold; quantified gravimetrically) using a Versatile Aerosol Concentration Enrichment System (VACES) from ambient downtown Louisville air as published (Haberzettl et al., 2012; O'Toole et al., 2010). Exposures were performed during months of summer (June and July) and, for comparison, during winter (January) because season‐dependent toxicity has been demonstrated by others (Mirowsky et al., 2013). Body weight was measured throughout exposures (pre‐/post‐exposure). Mice were euthanized immediately after the final exposure, and body weight, as well as weights of organs including lungs, heart, and spleen, were measured. Isolated lungs and thoracic aorta were either used fresh for functional assays or were frozen for subsequent biochemical analyses.

### 2.2 Urine collection and acrolein metabolite

Immediately prior to HEPA‐ and charcoal‐filtered air exposure or CAP exposure, mice were weighed and briefly exposed to D‐glucose/saccharin solution (w/v; 3.0%/0.125%; Sigma) on the mouth (Conklin, Haberzettl, Lesgards, et al., 2009; Wood et al., 2001). After 6h air or CAP exposure, mice were placed singly per metabolic cage (Harvard Apparatus) with D‐glucose/saccharin solution to collect urine (in graduated cylinders inside 4°C water‐jacketed organ baths) for 3 h. After urine collection, mice were placed in home cages overnight with food and water per normal housing arrangements. Urine samples were centrifuged (600 × *g*, 5 min, 4°C; to pellet any feces and food particles), decanted, and stored at −80°C. The major metabolite of acrolein, 3‐hydroxypropylmercapturic acid (3HPMA), was quantified in urine by mass spectrometry (negative ion mode) as previously described (Conklin et al., 2017). To account for urinary dilution, values for urinary 3HPMA were normalized to urinary creatinine (mg/dL).

### 2.3 Vascular function

Thoracic aortas were assayed for vascular reactivity as described with modification (Conklin et al., 2009b). Briefly, two adjacent, 3–4 mm aortic rings per mouse (one cleaned of PVAT; one with PVAT intact) were hung on stainless steel hooks in water‐jacketed organ baths (37°C) in physiological salt solution (PSS, 119 mM NaCl, 4.7 mM KCl, 1.2 mM MgSO_4_·7H_2_O, 1.2 mM KH_2_PO_4_, 25 mM NaHCO_3_, 1.6 mM CaCl_2_, 11.1 mM glucose, pH 7.4) (Lohn et al., 2002) bubbled with 95% O_2_ and 5% CO_2_. Rings (≈1 g loading tension) were contracted twice with 100 mM potassium solution and re‐equilibrated to ≈1 g over 1.5 h.

For contractions, agonists (phenylephrine, PE; U46,619) were added in single or in cumulative (0.1 nM–10 µM) concentrations. For endothelium‐dependent relaxation, PE‐precontracted (10 µM) aortas were relaxed with cumulative concentrations of acetylcholine (ACh; 0.1 nM–10 µM). For endothelium‐independent relaxation, PE‐precontracted aortas were relaxed with a single (100 µM) or cumulative concentrations of sodium nitroprusside (SNP; 0.1 nM–100 µM). To assess the specific contribution of nitric oxide synthase (NOS) to PE function, L‐NAME (100 µM) was added to the bath after PE‐induced tension had plateaued. The resulting increased tension was “PE+L‐NAME tension” and a “PE Contraction Ratio” was calculated as follows: PE+L‐NAME Tension / PE Tension (Jin et al., 2021). To measure endogenous leptin‐dependent action, two aortic rings with PVAT intact per mouse were pre‐incubated (15 min) with either nothing (control PVAT) or with a superactive leptin antagonist (SLAN, 1 μg/ml; Protein Laboratories Rehovot (PLR), Rehovot, Israel) before subsequent agonist addition (e.g., PE, U46,619, ACh, SNP). Additionally, isolated PVAT and aorta were used to examine insulin signaling (100 nM insulin, 15 min; Humulin‐RP) as described (Haberzettl, O'Toole, et al., 2016).

### 2.4 Gene array and qRT‐PCR

*Gene expression array*: Total RNA was isolated from collected PVAT samples using the miRNeasy kit (Qiagen #73404) and RNA quality assessed on an Agilent BioAnalyzer 2100. Pooled samples for each treatment condition (n = 4 per treatment group) were used for cDNA synthesis using the RT^2^ first strand kit (Qiagen #330401). These samples were then used to screen a mouse adipogenesis array (84 genes; Qiagen #330231) as per the manufacturer's instructions. Results were analyzed by the ΔΔC_T_ method (Table S1). *qRT*‐*PCR*: RNA was isolated from PVAT using the RNeasy Lipid Tissue Mini kit (Qiagen, Valencia, CA). Quantitative real‐time PCR was performed using the primer sets as listed in Table 1 and interleukin‐6 (IL‐6) primer set (SA Bioscience, Qiagen, Valencia, CA) (Haberzettl, O'Toole, et al., 2016).

**TABLE 1:** Quantitative real‐time PCR was performed in PVAT using the following primer sets as described (Haberzettl, O'Toole, et al., 2016)

Target	Forward primer	Reverse primer
*Tnf*	5′‐GCATGATCCGCGACGTGGAA−3′	5′‐AGATCCATGCCGTTGGCCAG−3′
*Ccl3*	5′‐ACTGACCTGGAACTGAATGCCTGA−3′	5′‐ATGTGGCTACTTGGCAGCAAACAG−3′
*Ccl2*	5′‐ATGCAGGTCCCTGTCATG−3′	5′‐GCTTGAGGTGGTTGTGGA−3′
*Il1b*	5′‐CTCCATGAGCTTTGTACAAGG−3′	5′‐TGCTGATGTACCAGTTGGGG−3′
*Lep*	5′‐AAAGAACCTGAGCTGAGGGTGACA−3′	5′‐ATGCTAATGTGCCCTGAAATGCGG−3′
*Adipoq*	5′‐AGACCTGGCCACTTTCTCCTCATT−3′	5′‐AGAGGAACAGGAGAGCTTGCAACA−3′
*Pparg*	5′‐ACATAAAGTCCTTCCCGCTGACCA−3′	5′‐AAATTCGGATGGCCACCTCTTTGC−3′
*Fabp4*	5′‐ATGAAATCACCGCCAGACGACAGGA−3′	5′‐TGTGGTCGACTTTCCATCCCACTT−3′
*Rppo*	5′‐AGATTCGGGATATGCTGTTGGC−3′	5′‐TCGGGTCCTAGACCAGTGTTC−3′

### 2.5 Western blotting

Western blot analysis was performed with lysates of aorta and PVAT as described (Conklin et al., 2015; Haberzettl, McCracken, et al., 2016). Briefly, collected tissues were lysed in RIPA buffer (50 mM Tris•HCl, pH 7.4; 150 mM NaCl; 1 mM EDTA; 0.25% sodium desoxycholate; 1% NP‐40; 1:100 protease inhibitor cocktail, Pierce, Rockford, IL; 1:100 phosphatase inhibitor, Sigma‐Aldrich) and proteins were separated by SDS‐PAGE and transferred to PVDF membranes (Bio‐Rad, Hercules, CA). Membranes were probed with antibodies against phospho‐Akt (Ser473) and Akt (1:1000; Cell Signaling Technology, Danvers, MA), or protein–acrolein adducts, and then developed using ECL® plus reagent (Amersham Biosciences, Piscataway, NJ). Detected band intensities (Typhoon 9400 variable mode imager, Amersham Biosciences) were quantified using Image Quant TL software (Amersham Biosciences) and normalized either to appropriate loading controls or total protein staining (amido black).

### 2.6 Biochemical analysis

Collected lungs were used to measure thiobarbituric acid reactive substances (TBARS) as described (Wheat et al., 2011).

### 2.7 Calculations and Statistics

Relaxation efficacy was calculated as a percentage reduction of agonist‐induced tension. Agonist‐induced contraction was normalized to aortic ring length (mN/mm). The effective concentration producing 10% (EC_10_) or 50% response (EC_50_) was assessed for each agonist by normalizing cumulative concentration responses to 100% response, plotting the % response versus the log [molar]_agonist_, with subsequent interpolation of EC_10_ and EC_50_. The pD_2_ was calculated as the –log(EC_50_).

Data are presented as mean ± SE. Statistical comparison for two groups was made using unpaired *t*‐test or Mann–Whitney U‐test (e.g., clean vs. PVAT). For comparisons with multiple groups, ANOVA on Ranks (Kruskal–Wallis test) with Bonferroni post hoc test was used (SigmaStat, SPSS, Chicago, IL). *p *< 0.05 was considered statistically significant.

## 3 RESULTS

### 3.1 PVAT structure and function

**FIGURE 1:**
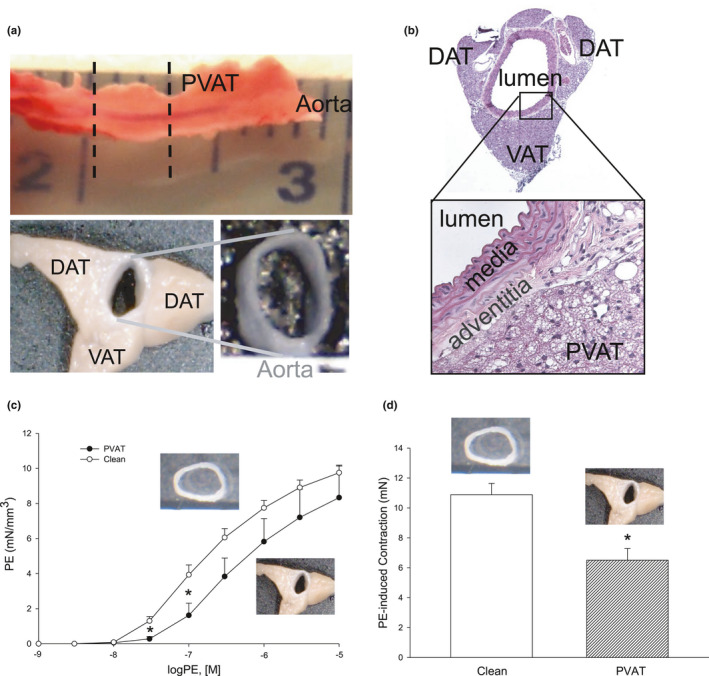
Murine thoracic aortic perivascular adipose tissue (PVAT) structure and function. a) Isolated thoracic aorta with PVAT intact (accompanying dashed lines indicate the approximate location of a 3‐mm segment to be used for functional assay). b) Image of a hematoxylin‐eosin stained cross‐section of the formalin‐fixed aorta with PVAT intact (40x magnification). *Inset*: Enhanced view of the interface between aortic wall adventitia and PVAT (400x magnification). c) Summary data of cumulative concentration‐dependent phenylephrine‐ (PE) induced increase in isometric tension (mN/mm^3^) of isolated aortic rings with and without PVAT. d) Summary data of the endogenous anti‐contractile effect of PVAT in aortas stimulated with PE (100 nM). Data are mean ± SE. n = 3. ^*^ *p* < 0.05 versus matched clean aorta

Vascular function was measured in adjacent sections of the isolated thoracic aorta that either had PVAT intact or was cleaned free of PVAT (Figure 1a). The aorta is surrounded by PVAT with three distinct components: two contiguous dorsal adipose tissue (DAT) flanges and a single triangle‐shaped ventral adipose tissue (VAT) portion (Figure 1a,b). Histological examination indicated that mouse thoracic aorta PVAT was largely BAT. As reported (Lohn et al., 2002), intact PVAT significantly damped PE‐induced increase in isometric tension (mN/mm^3^) of isolated aortic rings (Figure 1c,d) confirming the inherent anti‐contractile function of PVAT.

### 3.2 CAP exposure, PVAT, oxidative stress, and insulin resistance

**FIGURE 2:**
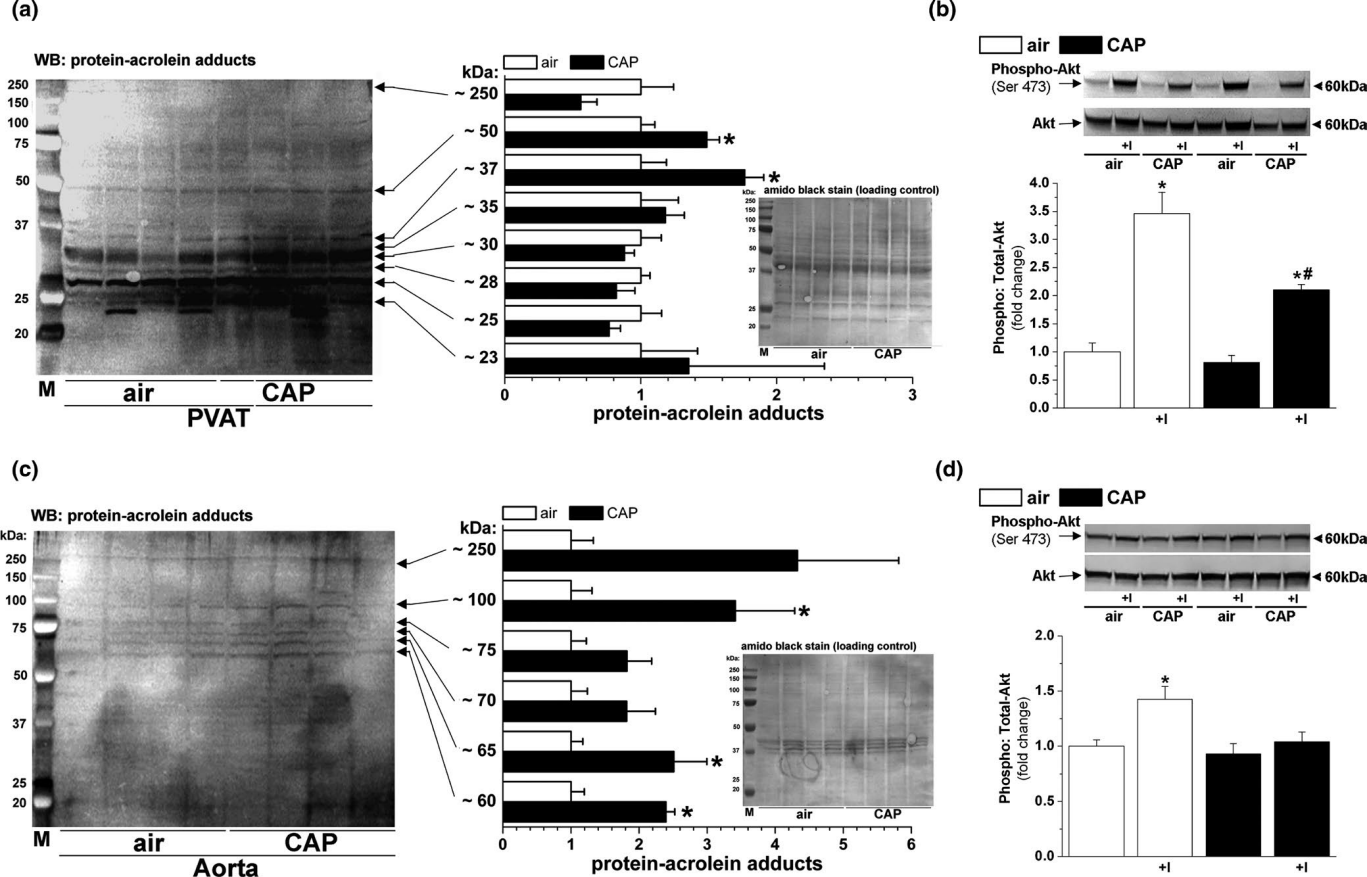
CAP‐induced PVAT oxidative stress and insulin resistance. Western blots and densitometric analyses of protein–acrolein adduct abundance in PVAT (a) and aorta (c) of mice exposed for 9 days to either air or CAP. Inset: Membranes stained with amido black served as loading controls. Data are mean ± SE normalized to air controls (n = 4, ^*^ *p* < 0.05 vs. air group). Insulin‐stimulated (+I, 100 nM, 15 min) Akt phosphorylation in isolated PVAT (b) and aorta (d) of mice exposed for 9 days to either air or CAP. Data are mean ± SE normalized to controls (n = 4; ^*^ *p* < 0.05 insulin vs. control; ^#^ *p* < 0.05 air vs. CAP)

As in our previous studies (Haberzettl et al., 2012; Haberzettl, O'Toole, et al., 2016), no overt systemic toxicity (e.g., no effect on the body, organ weight; Table 2) was observed in CAP‐exposed mice compared with air controls. Nonetheless, a 9‐day CAP exposure increased the abundance of protein–acrolein adducts (an oxidative stress marker) of 2–3 different molecular weight bands in both PVAT and aorta compared with matched air control organs (Figure 2a,c), indicating that CAP exposure induced oxidative stress at both sites. Specifically, CAP exposure increased the intensity of protein bands at molecular weights of 50 and 37 kDa in the PVAT (Figure 2a) and the band intensities of protein–acrolein adducts at 100, 65, and 60 kDa in the aorta (Figure 2c). Increased oxidative stress was accompanied by decreased insulin sensitivity in both isolated PVAT (Figure 2b) and aorta (Figure 2d). For example, CAP exposure significantly reduced insulin‐induced Akt phosphorylation in PVAT (air, 3.46 ± 0.34‐fold; CAP, 2.10 ± 0.10‐fold; Figure 2b). Similarly, CAP exposure impaired insulin‐induced Akt phosphorylation in cleaned aortas isolated from the same mice (Figure 2D) as observed in earlier studies (Haberzettl, McCracken, et al., 2016; Haberzettl, O'Toole, et al., 2016).

**TABLE 2:** Body and body:organ weight ratios in WT and ecSOD‐Tg mice exposed for 9 days to air or to CAP

Outcome	WT		ecSOD‐Tg	
	Air	CAP	Air	CAP
Body Weight (BW)	30.0 ± 0.1	29.5 ± 0.7	31.0 ± 0.7	31.0 ± 0.6
Lung:BW ratio (%)	0.55 ± 0.03	0.43 ± 0.11	0.53 ± 0.01	0.53 ± 0.01
Heart:BW ratio (%)	0.52 ± 0.02	0.47 ± 0.02	0.53 ± 0.02	0.47 ± 0.02
Spleen:BW ratio (%)	0.32 ± 0.03	0.23 ± 0.06	0.36 ± 0.04	0.32 ± 0.01

Values are mean ± SEM; n = 7–12.

Abbreviations: CAP, concentrated ambient fine particulate matter; ecSOD, extracellular superoxide dismutase; Tg, transgenic; WT, wildtype.

### 3.3 PVAT Leptin

**FIGURE 3:**
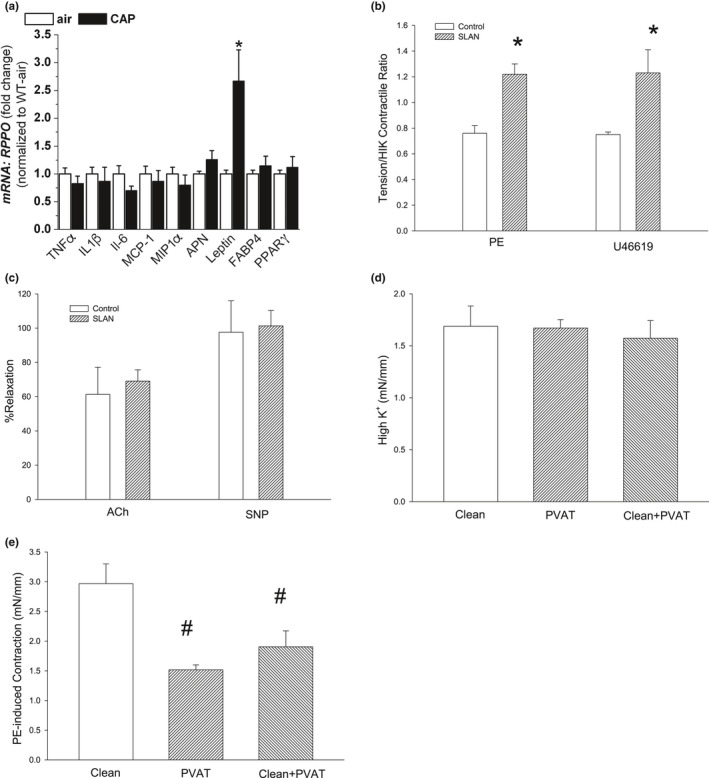
Gene expression and function of aortic PVAT. a) Relative abundance of inflammatory and adipose‐related mRNAs measured by *qRT*‐PCR analysis in PVAT isolated from WT mice exposed to either air or CAP for 9 days. b) Effect of the leptin receptor antagonist (SLAN) on aorta contractile response to PE and thromboxane A_2_ analog, U46,619. c) Effect of SLAN on aortic relaxation of ACh and SNP in PE‐precontracted aorta. d) Effect of PVAT anti‐contractile activity against 100 mM HI K^+^‐induced tension. e) PE‐induced tension (10 μM) in the clean aorta, aorta with intact PVAT, and clean aorta with isolated PVAT immersed in organ bath. Data are mean ± SE. ^*^ *p* < 0.05 versus air group (n = 4–8) or matched clean aorta (n = 3); ^#^0.10 > *p* > 0.05 versus matched clean aorta (n = 3)

To explore potential CAP‐induced transcriptional changes in PVAT, mRNA levels of adipogenic and adipokine genes were measured in PVAT by gene array. In the gene array, mRNAs of 20 genes (out of 84) were highly altered (14 increased; 6 decreased; Table S1). We found that the transcription of several genes was altered, which may portend long‐term differentiation of PVAT structure and function due to chronic CAP exposure. Interestingly, we also observed increased mRNA levels of two secreted adipokines (leptin, resistin) (Table S1). We confirmed the upregulation of *Lep* transcription in PVAT by *qRT*‐*PCR* (Figure 3a). No other adipokine or cytokine was increased in PVAT of CAP‐exposed mice.

Because CAP exposure increased *Lep* mRNA in PVAT, we investigated whether endogenous leptin was bioactive. For this, we used a superactive leptin receptor antagonist (SLAN) to block the activity of endogenous leptin. SLAN pretreatment in the isolated aorta of naïve mice significantly increased subsequent PE contraction (165 ± 22% of control; Figure 3b) and similarly increased U46,619‐induced tension (163 ± 18% of control; Figure 3b) compared with its paired aortic ring with PVAT. These data demonstrate an intrinsic anti‐contractile function of PVAT‐derived leptin. SLAN pretreatment had no effect on either endothelium‐dependent (ACh: 128 ± 30% of control) or ‐independent (SNP: 110 ± 17% of control) relaxations (Figure 3c). These data further support an intrinsic anti‐contractile effect of PVAT‐derived leptin. We also tested whether the anti‐contractile function of PVAT was dependent on direct contact (intact PVAT) with aorta. Intact PVAT or isolated PVAT placed adjacently in the bath had no effect on high K^+^ induced contraction (Figure 3d); however, both intact PVAT and isolated and adjacent PVAT modestly inhibited PE‐induced (10 μM) contraction (Figure 3e) showing that a freely diffusible, anti‐contractile agent (perhaps leptin) was released from PVAT.

### 3.4 Aorta and PVAT dysfunction

**FIGURE 4:**
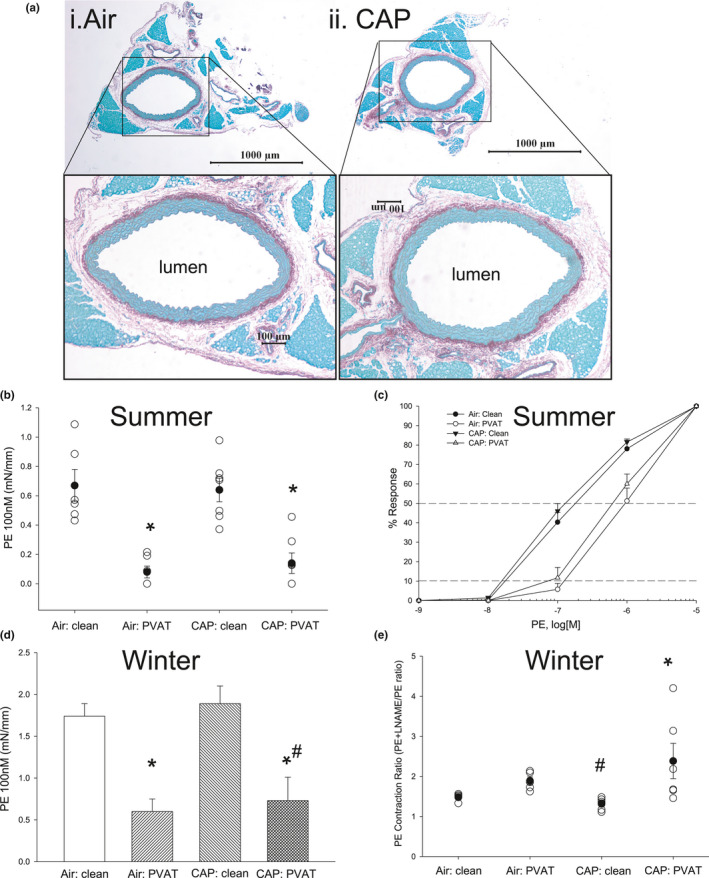
CAP‐induced effects on aorta and PVAT function. a) Representative images of aortic cross‐sections stained with Sirius Red/Fast Green (lamellae/collagen fibers and adventitia in red; PVAT and smooth muscle in green) of air‐ (Ai) and CAP‐ (Aii) exposed mice. b–d) Summary effects of 9‐day air and CAP exposures on responses of isolated aorta with and without PVAT to phenylephrine (efficacy and sensitivity) in (b, c) summer or (d) winter. (e) Summary data of “PE Contraction Ratio” (tension_PE+L‐NAME_/tension_PE alone_) of isolated aorta after 9‐day air and CAP exposures in winter. Data are mean ± SE. n = 4–8. * *p* < 0.05 versus matched clean aorta; ^#^0.10 > *p* > 0.05 versus matched air control

CAP exposure did not cause any obvious gross morphological changes in PVAT and aorta (Figure 4a) or aortic wall dimensions (data not shown), yet CAP altered aortic contractile and relaxation functions (Tables 3, 4, 5; Figure 4). Because PVAT exerted its strongest anti‐contractile effects at the near threshold concentration of PE (100 nM) rather than at maximal PE (10 μM), we quantified PE‐induced tension at 100 nM as well as at the effective PE concentration producing 10% (EC_10_) and 50% (EC_50_) of maximal tension. As expected, in air‐exposed WT mice, PVAT suppressed aortic contractility at 100 nM PE (Figure 4b,d) and PE sensitivity (indicated by a 6–7 X rightward shift in both the EC_10_ and the EC_50_; Figure 4c) in summer and in winter exposures despite similar levels of CAP (Tables 3, 4). For the most part, CAP exposures (9 days) did not alter the PVAT‐dependent directional (i.e., rightward) shift in PE contractility or its sensitivity, yet CAP exposure shifted the PE response curve of both clean and PVAT intact aorta to the left (i.e., increased sensitivity; Figure 4c). In fact, CAP marginally increased PE contractility (Figure 4b,d) and the PE EC_10_ (albeit slightly more in winter exposure) (Tables 3, 4). Supporting these notably subtle changes in both aortic and PVAT sensitivities to PE following CAP exposure, we observed a significant effect of winter CAP exposure on the PE Contraction Ratio (Figure 4e; Table 4). Because PE‐induced contractions are modulated by relaxing factors released from the endothelium and the PVAT, the PE Contraction Ratio (i.e., Tension_PE+L‐NAME_/Tension_PE_) quantifies how much modulation is due to nitric oxide synthase (NOS). Surprisingly, PVAT‐containing aorta of CAP‐exposed mice had the highest PE Contraction Ratio of all groups and it was significantly greater than in its matched clean aorta (Figure 4e; Table 4). In concert with these data, the clean aorta of CAP‐exposed mice had the lowest PE Contraction Ratio and was nearly significantly different from the PE Contraction Ratio of clean aorta of air‐exposed mice (Table 3; Figure 4e) indicating both a loss of endothelial‐derived NO and a PVAT‐derived compensatory source of NO.

**TABLE 3:** Perivascular adipose tissue (PVAT) dysfunction in the aorta of WT male mice exposed for 9 days to air or CAP (mean 66.6 µg/m^3^; range 50.0–81.0 µg/m^3^) during summer

Measurement	Air: clean	Air: PVAT	CAP: clean	CAP: PVAT
Aortic Length (mm)	3.47 ± 0.20	3.83 ± 0.22	3.56 ± 0.17	3.77 ± 0.13
HI K^+^ (100 mM)^1^	1.16 ± 0.15	1.31 ± 0.13	0.92 ± 0.06	1.06 ± 0.07
PE (100 nM)^1^	0.67 ± 0.11	0.08 ± 0.04*	0.64 ± 0.08	0.14 ± 0.07*
PE EC_10_ (nM)^2^	26 ± 5	166 ± 39*	18 ± 1	114 ± 28*
PE EC_50_ (nM)^3^	230 ± 65	1193 ± 214*	129 ± 19	754 ± 161*
PE pD_2_ ^4^	6.77 ± 0.11	5.96 ± 0.08*	6.92 ± 0.07	6.19 ± 0.11*
ACh (% relax, PE)^5^	−42.9 ± 3.8	−51.3 ± 8.4	−53.9 ± 5.8	−53.3 ± 9.0
ACh EC_50_ (µM)^3^	1.59 ± 0.64	1.59 ± 0.62	0.86 ± 0.28	1.22 ± 0.42
ACh pD_2_ ^4^	6.09 ± 0.28	6.01 ± 0.21	6.29 ± 0.22	5.96 ± 0.31
SNP (% relax, PE)^5^	−102.5 ± 9.6	−123.1 ± 18.3	−114.4 ± 7.6	−119.5 ± 10.8

Data are mean ± SEM; n = 6–8 mice.

Abbreviations: ACh, acetylcholine; CAP, concentrated ambient particulate matter; HI K^+^, high potassium buffer; PE, phenylephrine; SNP, sodium nitroprusside; WT, wildtype.

^1^ mN/mm.

^2^ EC_10_, effective concentration producing 10% response (in nM or µM as indicated).

^3^ EC_50_, effective concentration producing 50% response (in nM or µM as indicated).

^4^ pD_2_, ‐log [EC_50_].

^5^ Relaxation as a percentage of agonist‐induced tension.

* *p* < 0.05 versus matched clean aorta (Mann–Whitney U‐test).

**TABLE 4:** Perivascular adipose tissue (PVAT) dysfunction in the aorta of WT male mice exposed for 9 days to air or CAP (54.6 µg/m^3^) during winter

Measurement	Air: clean	Air: PVAT	CAP: clean	CAP: PVAT
Aortic Length (mm)	3.35 ± 0.34	3.56 ± 0.33	3.68 ± 0.36	3.73 ± 0.37
HI K^+^ (100 mM)^1^	1.74 ± 0.39	1.96 ± 0.42^†^	1.77 ± 0.36	2.07 ± 0.56
PE (100 nM)^2^	1.74 ± 0.15	0.60 ± 0.15^†^	1.89 ± 0.21	0.73 ± 0.28*
PE EC_10_ (nM)^3^	12 ± 4	36 ± 6^†^	11 ± 4	37 ± 8*
PE EC_50_ (nM)^4^	124 ± 51	316 ± 104^†^	106 ± 46	218 ± 46
PE pD_2_ ^5^	7.08 ± 0.20	6.58 ± 0.13^†^	7.20 ± 0.20	6.71 ± 0.09^†^
PE Contraction Ratio^6^	1.48 ± 0.04	1.89 ± 0.10^†^	1.31 ± 0.06^#^	2.39 ± 0.44*
ACh (%, PE)^7^	−69.9 ± 3.3	−86.5 ± 3.6^†^	−67.9 ± 5.5	−85.3 ± 6.7
ACh EC_50_ (nM)^4^	92.9 ± 21.2	298.8 ± 200.2	176.2 ± 61.05	201.2 ± 67.5
ACh pD_2_ ^5^	7.09 ± 0.12	6.90 ± 0.29	6.94 ± 0.20	6.81 ± 0.14
SNP (%, PE)^7^	−99.8 ± 1.2	−101.7 ± 0.9	−107.7 ± 3.8	−108.0 ± 3.2
SNP EC_50_ (nM)^4^	8.48 ± 1.52	70.81 ± 41.42^†^	8.69 ± 1.63	64.73 ± 22.06*
SNP pD_2_ ^5^	8.10 ± 0.08	7.44 ± 0.25^†^	8.10 ± 0.08	7.33 ± 0.17*

Data are mean ± SEM; n = 6–8 mice.

Abbreviations: ACh, acetylcholine; CAP, concentrated ambient particulate matter; HI K^+^, high potassium buffer; PE, phenylephrine; SNP, sodium nitroprusside; WT, wildtype.

^1^ mN/mm.

^2^ EC_10_, effective concentration producing 10% response (in nM or µM as indicated).

^3^ EC_50_, effective concentration producing 50% response (in nM or µM as indicated).

^4^ pD_2_, ‐log [EC_50_].

^5^ SNP‐induced [100 μM] relaxation as a percentage of the indicated contractile agonist‐induced tension.

^6^ L‐NAME + PE/PE.

^7^ Relaxation as a percentage of agonist‐induced tension.

* *p* < 0.05 versus matched clean aorta (Mann–Whitney U‐test).

^†^ 0.10 > *p* > 0.05 versus matched clean aorta (Mann–Whitney U‐test).

# *p* < 0.05 air versus CAP (unpaired *t*‐test).

**TABLE 5:** Role of perivascular adipose tissue (PVAT) in vascular dysfunction of the isolated aorta from ecSOD‐Tg mice exposed for 9 days to air or CAP (mean 66.6 µg/m^3^; range 50.0–81.0 µg/m^3^) during summer

Measurement	Air: clean	Air: PVAT	CAP: clean	CAP: PVAT
Aortic length (mm)	3.41 ± 0.16	3.86 ± 0.14	3.66 ± 0.19	4.09 ± 0.20
HI K^+^ (100 mM)^1^	0.94 ± 0.14	1.15 ± 0.10	0.81 ± 0.08	1.10 ± 0.10
PE (100 nM)^1^	0.66 ± 0.11	0.15 ± 0.11*	0.73 ± 0.10	0.04 ± 0.02*
PE EC_10_ ^2^	20 ± 5	272 ± 79*	19 ± 3	279 ± 77*
PE EC_50_ ^3^	170 ± 57	1683 ± 343*	110 ± 23	1543 ± 323*
PE pD_2_ ^4^	6.88 ± 0.13	5.83 ± 0.10*	7.02 ± 0.09	5.90 ± 0.11*
ACh (% relax, PE)^5^	−39.1 ± 7.0	−53.1 ± 8.2	−33.8 ± 7.3	−56.2 ± 3.4^†^
ACh EC_50_ (µM)^3^	2.33 ± 0.88	1.37 ± 0.42	1.34 ± 0.55	3.35 ± 0.96
ACh pD_2_ ^4^	5.96 ± 0.31	5.98 ± 0.14	6.02 ± 0.18	5.63 ± 0.23
SNP (% relax, PE)^5^	−97.1 ± 12.3	−106.5 ± 7.6	−88.8 ± 4.5	−111.2 ± 9.3

Data are mean ± SEM; n = 5–8 mice.

Abbreviations: ACh, acetylcholine; CAP, concentrated ambient particulate matter; HI K^+^, high potassium buffer; PE, phenylephrine; SNP, sodium nitroprusside.

^1^ mN/mm.

^2^ EC_10_, effective concentration producing 10% response (in nM or µM as indicated).

^3^ EC_50_, effective concentration producing 50% response (in nM or µM as indicated).

^4^ pD_2_, ‐log [EC_50_].

^5^ Relaxation as a percentage of agonist‐induced tension.

* *p *< 0.05 versus matched clean aorta (Mann–Whitney U‐test).

^†^ 0.10 > *p *> 0.05 versus matched clean aorta (Mann–Whitney U‐test).

As in a previous study of short‐term CAP exposure (Haberzettl, O'Toole, et al., 2016), we did not observe obvious frank endothelial dysfunction in the cleaned aorta after CAP exposures (summer or winter) (Tables 3, 4). Nonetheless, the effect of PVAT on ACh‐induced relaxation (%), although noticeably increasing the overall magnitude (efficacy) of relaxation, did not reach statistical significance, despite trending this way in air‐exposed mice in winter (Table 4). Likewise, PVAT did not enhance or reduce the sensitivity of the aorta to ACh‐induced relaxation as measured by EC_50_ and pD_2_ (Tables 3, 4). Similarly, CAP exposure did not appear to alter the aortic response to the vasorelaxant NO donor, sodium nitroprusside (SNP), whether quantified as efficacy (absolute % relaxation; Tables 3, 4) or sensitivity (EC_50_; Table 4 only). However, PVAT did dampen modestly (0.10 > *p *> 0.05) the aortic response to SNP as reflected in a 3‐fold rightward shift in the EC_50_ in air‐exposed mice (Table 4), while CAP (winter exposure) intensified this PVAT‐dependent inhibitory effect to a statistically significant level (Table 4).

### 3.5 Role of pulmonary oxidative stress in CAP‐induced vascular dysfunction

**FIGURE 5:**
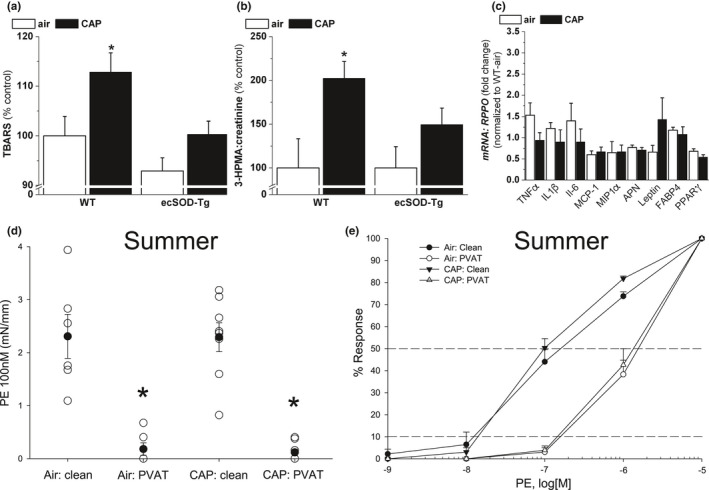
Prevention of CAP‐induced effects in ecSOD‐Tg mice. a) Levels of thiobarbituric acid reactive substances (TBARS) measured in the lungs of ecSOD‐Tg mice and their WT littermates inhaling air or CAP for 9 days. b) Urinary levels of the acrolein metabolite 3‐hydroxypropylmercapturic acid (3HPMA) in WT and ecSOT‐Tg mice exposed for 6 h to air or CAP. c) Relative abundance of inflammatory and adipose‐related mRNAs measured by *qRT*‐PCR in PVAT isolated from ecSOD‐Tg mice inhaling either air or CAP. The *qRT*‐PCR data are normalized to the data obtained from the WT‐air controls of the same exposure experiments that are shown in Figure 3a. Data are mean ± SE (n = 3–9; *p < 0.05 vs. matched air control). d–e). CAP exposure had no effect on anti‐contractile function of PVAT to single concentration (100 nM; d) or cumulative PE (1–1,000 nM; e) in ecSOD‐Tg mice. Data are mean ± SE (n = 4–8; *p < 0.05 vs. matched clean aorta)

Because CAP increased oxidative stress markers (acrolein–protein adducts) in PVAT and aorta, we asked whether CAP similarly increased pulmonary and systemic markers of oxidative stress. Consistent with this idea, we measured increased levels of lung TBARS (Figure 5a) and urinary 3HPMA (major acrolein metabolite, 6 h, 280 μg/m^3^; Figure 5b) in CAP‐exposed mice versus air control mice. To demonstrate these changes were related to each other, and, also with vascular effects, we exposed ecSOD‐Tg mice to air and CAP for 9 days and measured oxidative stress markers and PVAT genes and aortic function. Overexpression of ecSOD in the lungs not only prevented pulmonary and systemic oxidative stress markers (Figure 5a,b), but also prevented changes in *Lep* mRNA in PVAT of CAP versus air‐exposed ecSOD‐Tg mice (Figure 5c). Similarly, aortic and PVAT functional responses were indistinguishable between air‐ and CAP‐exposed ecSOD‐Tg mice (Figure 5d,e), excepting a slight enhancement of ACh‐induced relaxation by PVAT compared with clean aorta in CAP‐exposed ecSOD‐Tg mice (Table 5). Overall, these data show that CAP induces pulmonary oxidative stress leading to alterations in PVAT *Lep* gene expression and vascular dysfunction.

## 4 DISCUSSION

Our study examined the unknown role of aortic PVAT in the vascular dysfunction induced by short‐term air pollution exposure. Recent studies implicate an important role of PVAT in the development of coronary artery disease through a process termed “outside‐in” remodeling. This process contends that pro‐inflammatory changes in the PVAT that surrounds conduit arteries precede and cause deterioration in vascular function including the onset of endothelial dysfunction—*sine qua non* of atherosclerotic disease (Blomkalns et al., 2010). Although this idea is well established in diet‐induced obesity studies (Owen et al., 2013; Payne et al., 2012), limited data exist regarding the effects of environmental determinants such as air pollution on PVAT’s contribution to vascular dysfunction (Kampfrath et al., 2011). Because “cause and effect” are impossible to disentangle in chronic studies, we opted for a short‐term (9‐day) exposure of mice to CAP (downtown Louisville air) to investigate PVAT and vascular changes. Previous studies (under these conditions) show that short‐term CAP induces aortic vascular inflammation and aortic insulin and VEGF resistance in the absence of frank endothelial dysfunction (Haberzettl et al., 2012; Haberzettl, McCracken, et al., 2016; Haberzettl, O'Toole, et al., 2016). Similarly, herein, we detect early (albeit subtle) changes in PVAT and aortic function that complement our previous findings, yet clearly, emphasize the complex, intertwined relationship between PVAT changes and their effects on aortic function. Novel changes found in vascular tissue after short term CAP exposure include: (a) increased PVAT *Lep* mRNA but not inflammatory genes; (b) increased PVAT anti‐contractile function and aortic contractility; (c) increased PVAT, aortic, and systemic oxidative stress related to lipid peroxidation and subsequent acrolein formation; (d) induced PVAT insulin resistance; and, (e) prevention of CAP‐induced effects on PVAT and aorta in mice with pulmonary overexpression of ecSOD (ecSOD‐Tg mice).

The specific mechanisms that lead to these early aortic and PVAT alterations appear to depend on coordinated changes in the lungs (i.e., pulmonary oxidative stress) and vascular PVAT (increased oxidative stress and *Lep* transcription)—a conclusion that is supported by showing ecSOD‐Tg mice are protected from CAP exposure‐induced effects. These data are consistent with the findings of Kampfrath *et al*., who demonstrate that chronic exposure of mice to concentrated PM_2.5_ led to increases in oxidative stress in lungs and PVAT—both effects being dependent on toll‐like receptor‐4 (TLR4) and NADPH oxidase (Nox) (Kampfrath et al., 2011). Our current study furthers this narrative by showing that early changes in leptin transcription in PVAT are concurrent with dynamic alterations in vascular reactivity albeit not endothelial dysfunction *per se*. These data argue that PVAT‐derived *Lep* may be an early sign of either injury or compensation. Perhaps, any changes in PVAT‐derived *Lep* (and other up or downregulated factors as detected by gene array) portend future changes in vascular function, including frank endothelial dysfunction, after chronic exposure to air pollution.

In the short term, leptin may be acting as a compensatory signal for loss of endothelium function (albeit a subtle loss) seen as decreased NO (EDRF) release/bioavailability in PVAT‐free blood vessels (Campen, 2009). Several observations support this conclusion. One, *Lep* mRNA increases following CAP exposure. Two, leptin plays an important endogenous role in the anti‐contractile activity of PVAT. For example, the novel leptin receptor antagonist (mutated leptin; SLAN) enhances agonist‐induced contractions without altering ACh or SNP relaxations. It is well known that multiple adipose‐derived relaxing factors (ADRF) are released from PVAT including adiponectin (Hou et al., 2016). Notably, in our current study, CAP exposure only modestly changed PVAT *Retn* and *Adipoq* mRNA. Third, the CAP‐induced change in *Lep* mRNA is absent in ecSOD‐Tg mice exposed to CAP as are modest alterations in vascular function. Finally, the most compelling “compensatory action” of PVAT is present in the aorta with PVAT of CAP‐exposed mice. The addition of L‐NAME to PE‐precontracted aorta (with PVAT intact) increases tension dramatically (i.e., PE Contraction Ratio) compared with clean aorta (see Figure 4d). We infer that these data indicate PVAT changes may be compensatory, in part, to enhance NO production via NOS following CAP exposure. Addition of a NOS inhibitor, for example, L‐NAME, in the presence of PE, increases tension in proportion to real‐time NO generated via NOS (Jin et al., 2021). Thus, as the “PE Contraction Ratio” increases, it reflects greater contribution of NO, and furthermore, in the clean aorta, it implicates selectively the endothelium as the source of NO (eNOS). Yet, in thoracic aorta with PVAT intact, modulation of PE contraction may derive multiple NOS sources (e.g., eNOS, nNOS, and iNOS), which are all blocked by L‐NAME, although thoracic aorta PVAT contains eNOS (Victorio et al., 2016). Nonetheless, this phenomenon is not selective for CAP exposure, as it is previously reported with high‐fat diet feeding (Gil‐Ortega et al., 2010). Also, CAP exposure induces PVAT insulin resistance, accumulation of PVAT protein–acrolein adducts and increases urinary 3HPMA (major metabolite of acrolein) indicating both PVAT and aorta are simultaneously under the assault of oxidative stress (Kampfrath et al., 2011; Ying et al., 2009). We previously showed that exogenous acrolein induces insulin resistance in endothelial cells (O'Toole et al., 2014), which adds plausibility that CAP‐induced “*acrolein stress*” may mediate these local effects. Future studies are needed to disentangle “injurious vs compensatory” PVAT, leptin, and vascular responses.

Although PVAT‐derived *Lep* may be compensatory in the short term, chronic exposure to leptin in the vascular wall likely leads to a more dysfunctional aorta with phenotypically altered vascular smooth muscle cells (VSMC) primed both for the proliferation and promotion of atherosclerotic plaque formation (Noblet et al., 2016). VSMC undergo a well‐described “phenotypic switch” with growth factor stimulation that decreases smooth muscle α‐actin and calponin with increases in osteopontin (Salabei et al., 2013). Thus, CAP exposure as with other stressors (e.g., high‐fat diet feeding) augments an underlying process that may converge on a common pathophysiological pathwaymediated, in part, by PVAT‐derived leptin (Knudson et al., 2008). It is well documented that chronic PM_2.5_ exposure induces endothelial dysfunction, adipose inflammation, insulin resistance, and atherosclerosis especially in conjunction with high‐fat diet (Deiuliis et al., 2011; Sun et al., 2005, 2009). Potentially, intervention with selective leptin receptor antagonists may be beneficial as proposed (Payne et al., 2014). Revealing the mediator that upregulates PVAT leptin (e.g., acrolein?) following PM exposure (or high‐fat diet) is critical to better understand this process. For example, a recent metabolomics study shows that short‐term CAP exposure leads to a dose‐dependent alteration in the plasma lipidome including free fatty acids, such as, palmitate, that is largely prevented in ecSOD‐Tg mice suggesting these changes may be causal (or coincident) with other systemic changes (Hill et al., 2021). Thus, more data are required before beginning any stage‐specific, targeted intervention of PVAT dysfunction in order to prevent inhalation‐associated vascular diseases (Kim et al., 2020).

## DISCLOSURES

The authors have no perceived or potential conflicts of interest to disclose.

## AUTHOR CONTRIBUTIONS

P.H., L.J., J.Z, T.O., and D.J.C. designed and performed experiments and data analyses, contributed to discussions, wrote, and edited the manuscript. D.W.R assisted in statistical analyses. D.J.C is the guarantor of this work and as such had full access to all the data in the study and takes responsibility for the integrity and the accuracy of the data and its analyses.

## Supporting information



Supplementary Material
