# Supplementary material for: Fine particulate matter air pollution and aortic perivascular adipose tissue: Oxidative stress, leptin, and vascular dysfunction
Source: Physiol Rep. 2021 Jul 29;9(15):e14980. doi: 10.14814/phy2.14980 (PMC8322754; doi:10.14814/phy2.14980)
Supplement: Supplementary file 1 — Supplementary Material [file PHY2-9-e14980-s001.docx]

**Supplemental Table 1: Gene array results of highly altered mRNA levels in perivascular adipose tissue (PVAT) of mice exposed for 9-days to concentrated ambient fine particulate matter (CAP) vs HEPA-filtered air-exposed control mice.**

| **Increased** | | |
| --- | --- | --- |
| **Protein** | **Gene** | **Air vs CAP: 2^(-ΔΔCT)^** |
| Beta-2 adrenergic receptor | *Adrb2* | 2.536 |
| Bone morphogenic protein 4 | *Bmp4* | 1.478 |
| Bone morphogenic protein 7 | *Bmp7* | 1.575 |
| Forkhead box C2 | *Foxc2* | 1.932 |
| Insulin receptor substrate 2 | *Irs2* | 1.603 |
| Krüppel-like Factor 2 | *Klf2* | 5.336 |
| Leptin | *Lep* | 1.520 |
| Nuclear receptor co-repressor 2 | *Ncor2* | 2.078 |
| Nuclear receptor subfamily 0, group B, member 2 | *Nr0b2* | 1.763 |
| Resistin | *Retn* | 1.493 |
| Sirtuin 1 | *Sirt1* | 1.553 |
| Rous sarcoma oncogene | *Src* | 1.588 |
| TSC22 domain family, member 3 | *Tsc22d3* | 1.538 |
| Vitamin D receptor | *Vdr* | 3.870 |
| **Decreased** | | |
| **Protein** | **Gene** | **Air vs CAP: 2^(-ΔΔCT)^** |
| Adipogenin | *Adig* | 0.668 |
| Dickkopf homolog 1 | *Dkk1* | 0.604 |
| Nuclear receptor subfamily 1, group H, member 3 | *Nr1h3* | 0.641 |
| Peroxisome proliferator activated receptor alpha | *Ppara* | 0.687 |
| Uncoupling protein 1 | *Ucp1* | 0.622 |
